# The Contribution of Hydrophobic Interactions to Conformational Changes of Inward/Outward Transmembrane Transport Proteins

**DOI:** 10.3390/membranes12121212

**Published:** 2022-11-30

**Authors:** Irena Roterman, Katarzyna Stapor, Leszek Konieczny

**Affiliations:** 1 Department of Bioinformatics and Telemedicine, Jagiellonian University—Medical College Medyczna 7, 30-688 Kraków, Poland; 2 Department of Applied Informatics, Faculty of Automatic, Electronics and Computer Science, Silesian University of Technology, Akademicka 16, 44-100 Gliwice, Poland; 3 Chair of Medical Biochemistry—Jagiellonian University—Medical College, Kopernika 7, 31-034 Kraków, Poland

**Keywords:** transmembrane proteins, outward, inward, symport/antiport transport, antibiotic resistance

## Abstract

Proteins transporting ions or other molecules across the membrane, whose proper concentration is required to maintain homeostasis, perform very sophisticated biological functions. The symport and antiport active transport can be performed only by the structures specially prepared for this purpose. In the present work, such structures in both In and Out conformations have been analyzed with respect to the hydrophobicity distribution using the FOD-M model. This allowed for identifying the role of individual protein chain fragments in the stabilization of the specific cell membrane environment as well as the contribution of hydrophobic interactions to the conformational changes between In/Out conformations.

## 1. Introduction

Membrane proteins (including transmembrane proteins—TM) responsible for the transport of molecules, including ions, in particular, play critical roles for the functioning of the cell. Their activity ensures that the concentrations of the appropriate ingredients are maintained at the level required for their proper functioning. The analysis of the transport mechanism as well as the structure and biological activity of transmembrane proteins is the subject of numerous studies [1,2,3,4,5,6,7,8,9,10,11]. The discussed phenomena concern passive diffusion and active transport. Passive diffusion consists in the penetration of certain specific molecules along a gradient leading to a higher entropic state as an effect of striving to equalize concentrations with the accompanying reduction of the enthalpy level [12]. This type of movement through the membrane concerns both O_2_ and CO_2_, which, due to their small size and non-polar nature, dissolve relatively easily in the membrane environment [13]. This process does not require the participation of proteins. Ion transport is not possible in this way of diffusion due to the impermeability of the cell membrane to charges [14].

Active transport involves the movement of materials *against* a concentration gradient and requires and expenditure of energy. The coupled transport of two distinct molecules is called co-transport. This type of transport carried out by a protein acting as a transporter takes various forms. If the two molecules are transported in the same direction, such transport is called symport [15,16], while antiport [17,18,19] is reserved for the transport in opposite directions. The movement of a single molecule is called uniport [20,21]. The term pumps refers to a transport process that requires energy (in the form of ATP hydrolysis) resulting in the appearance of an electrochemical gradient that supports the transport process. A very important example of this is the sodium-potassium pump [22,23,24,25,26]. A detailed classification of the types of transport and the specificity of proteins carrying out this process are discussed in the Transporter Classification Database [27,28,29,30].

The structures of transmembrane proteins in both inward and outward conformations are available in the PDB database, which enables the assessment of transport-related structural changes [31]. Transmembrane protein contacting mainly with the hydrophobic membrane and the polar environment in the extra-membrane parts, shows a perfect adaptation to these different conditions, representing the hydrophobicity distribution referred to as amphiphilic or amphipathic [32]. 

In structuring membrane proteins, the hydrophobicity characterization within the amino acid sequence is of primary importance. It enables the adaptation to the mentioned, diverse environmental conditions [33,34,35,36,37,38,39,40,41,42,43,44,45,46]. 

The present work presents the analysis of the hydrophobicity distribution in transmembrane proteins using the fuzzy oil drop model (FOD) in its modified version (FOD-M [47,48,49]). The comparative analysis carried out in the present work is based on the tracking of changes in the hydrophobicity distribution in the symport/antiport proteins in both conformations (In/Out). By determining the status of individual chain fragments based on the parameters from the FOD-M model, it is possible to determine the contribution of hydrophobic interactions to the structural changes between In/Out conformations of these proteins.

## 2. Materials and Methods

### 2.1. Data

The object of the analysis are three symport transmembrane proteins and one antiport. The discussed proteins show the multi-pass topology, where the helical segments referred to as TM (transmembrane) form an up-down bundle. The description of the analyzed proteins is presented in Table 1.

### 2.2. The FOD/FOD-M Models

All life processes in living organisms require an aquatic environment. Hence, the hydrophobicity of molecules present in the cell’s environment is very important and is the subject of many analyzes, discussions and studies [33,34,35,36,37,38,39,40,41,42,43,44,45,46]. 

In particular, the discrete two-layers model called the “oil drop” [56] (polar surface—hydrophobic center) was modified resulting in the fuzzy oil drop (FOD) [57] model, where the 3D Gaussian function fitted to the protein body represents a gradual decrease in the level of hydrophobicity from the center (maximum) to the surface (close to zero). The form of the 3D Gaussian function is adapted to the size and shape of the protein molecule by estimating the distribution parameters. The determined value of this function in the position of the effective atom (i.e., the average position of the atoms included in the amino acid)—as it is assumed—represents the idealized hydrophobicity distribution, the so-called expected or theoretical (*T*). Normalized *T* values thus represent a hydrophobicity distribution consistent with an idealized micelle. This distribution is confronted with the real/observed O distribution, which is the result of hydrophobic interactions between the residues. In FOD model, the function proposed in [58] was used.

The normalized *O* values can be compared with the corresponding *T* values, thus obtaining information on the hydrophobicity compatibility positions (expected and actual) in the protein. The degree of agreement (similarity) of the distributions is calculated in the FOD model using the probabilistic Kulback–Leibrer distance (D_KL_).

In order to compare the distributions in different proteins, a reference distribution—R was introduced in the FOD model, where each residue (effective atom) is assigned the same hydrophobicity level equal to 1/N where N is the number of residues in the protein.

The comparison of D_KL_(*O*|*T*) and D_KL_(*O*|*R*) “distances” is important because the *O* distribution is confronted here with a distribution with a perfectly constructed core (*T* distribution) and a distribution devoid of any hydrophobicity diversity (*R* distribution).

To avoid the need to operate with two values, the parameter *RD* (Relative Distance) was introduced in the FOD model, which expresses the relative distance D_KL_(*O*|*T*) to the sum of the distances D_KL_(*O*|*T*) + D_KL_(*O*|*R*). The value of *RD* < 0.5 means that the *O* distribution is similar to the *T* distribution, which is interpreted as the statement of the presence of a hydrophobic core. The value of the *RD* parameter also determines the degree of this approximation. It is possible to create a ranking list comparing the degree of nuclear order in e.g., homologous proteins.

Identification of the reasons for the divergence of the *O* distribution versus the T distribution—identification of the residues with the maximum divergence—allows for indicating the residues with a local excess and deficiency in hydrophobicity. The first type of discrepancy allows for the identification of the complexation site of another protein [48] and the second—ligand binding cavity [49].

Membrane proteins are characterized by a different type of environment compared to the aquatic environment. Here, the opposite to the centric distribution is expected. Exposure of hydrophobic residues on the surface is expected to stabilize contact with the hydrophobic membrane and a low level of hydrophobicity in the central part of the protein (where different types of channel are usually located). Therefore, the following function is used to describe the expected hydrophobicity distribution in the modified FOD-M model [59]:

$$M={\left[T+K{\left(T_{\mathrm{MAX}}-T\right)}_{n}\right]}_{n}$$

where *T* denotes the 3D Gaussian function spread over the data and (*T*_MAX_ − *T*)—the “inverted” function to the 3D Gaussian. The *K* parameter determines the degree—the force with which the force field of the water (polar) environment is modified by the field with the inverted characteristics (membrane), Index *n*—normalization.

The *M*-distribution is the representative of the external force field and the target distribution of a protein shaped by a non-polar environment. The membrane protein “restores” the *M* distribution by adapting to the environmental conditions. Water-soluble proteins are described with the value of the parameter *K* = 0 or in the range 0 < *K* < 0.4. Membrane proteins present the values of *K* > 0.9, even reaching *K* > 3 [59,60,61,62]. A detailed description of the discussed field is available in [59], where the application of the FOD-M model for different groups of membrane proteins is discussed.

The FOD-M model was used in this study to identify structural changes related to the transport of molecules and ions in the symport/antiport systems due to the availability of these forms of membrane proteins in PDB [31].

## 3. Results

The calculated *RD* and *K* parameters of the FOD model for the proteins in question are presented in Table 2.

The pairs of proteins listed in Table 2 were analyzed using the FOD-M model, using the *T*, *O* and *M* distributions for an appropriately selected value of the parameter *K*. The characteristics of these proteins show a pattern typical of membrane proteins with relatively high values of *RD* and *K* parameters compared to the analogous values for water-soluble proteins. This is due to the need to expose hydrophobic residues on the surface—ensuring stabilization in the environment of a hydrophobic membrane, and the absence of a centrally located hydrophobic nucleus, where in the case of transmembrane channels the channel is present—i.e., either free space or low packing of amino acids. This deviation from the micelle-like system necessitates a significant modification of the expected distribution (*T* distribution) for proteins operating in the hydrophobic environment of the membrane.

Despite slight differences in *RD* and *K* parameters for the Outward and Inward forms, a detailed analysis of the *T*, *O* and *M* profiles reveals the structural changes related to the transport of ions/molecules through the cell membrane. The 4ZP0/6GV1 pair shows unchanged status of outward and inward forms both from the *RD* and *K* point of view. It is a protein representing antiport activity. Since both directions for transport are possible, the inward/outward system should be on both sides of the transporter. Therefore, the comparable values of *RD* and *K* for this system do not come as a surprise. 

The biggest change in the comparison of Inward and Outward forms is shown by the pair **6E9N**/**6E9O**, which probably allows for unequivocal identification of structural changes by changing the status expressed by the values of *RD* and *K*. 

In contrast, the 5AYO/5AYM pair reveals the highest *K* values (>1.0). This means the highest adaptation to the membrane environment in the discussed group of proteins.

### 3.1. The Analysis of Individual Examples

Before the analysis, we present a few facts necessary in the interpretation of the constructed FOD-M model for the proteins in question. The failure to adjust the *O* and *T* distributions in the form of a hydrophobicity deficit (i.e., the local maximum in *T* distribution is absent in *O* distribution) may result generally from two reasons:

1. In the center of a protein there is a highly polar residue (for example lysine), which significantly reduces the level of hydrophobicity;
2. In the center of a protein is a free space. In this situation, even residues with a high level of hydrophobicity (due to the reduced number of other residues with which interaction is possible) cause a reduction in the value of the observed hydrophobicity *O* (which is the sum of interactions).

The difference between the two reasons is that the failure (mismatch) according to reason 1 above has the form of a single peak involving only one residue (e.g., the lysine mentioned).

In the proteins in question, reason 2 is the cause of the *O* distribution not being adapted to the *T* distribution (of course the reason 1 is not completely excluded). 

The change in the protein section’s status can be expressed by the difference in the values of parameter *RD*. This difference expresses the size of the divergence of the *O* profile in relation to the *T* one. On the other hand, the value of the parameter *K* expresses a measure of the contribution of a non-aquatic environment (contact) to obtain a given observed *O* distribution. 

In the present study, when identifying the change in the status of individual TMs in pairs, the change in parameter *K* was (mainly) taken into account.

Hereinafter, for the terms Inward and Outward, the abbreviations In and Out, respectively are used.

#### 3.1.1. Putative Bacterial Homologue of Ferroportin (BbFPN) 

This protein is represented by the structures PDB ID 5AYO (inward-facing) and 5AYM [50,63] (outward-facing). Despite the lack of similarity of their sequences to ferroportins, they are treated as its representatives due to the significant degree of similarity and belonging to the same type of folding [50].

The iron ion transport mechanism is here according to the symport scheme. The biological role of ferroportins is critical in maintaining iron homeostasis. Therefore, the recognition of the mechanism of action of ferroportins becomes important for therapeutic purposes, as the disturbance of the correctness of this transport results in *iron-deficiency anemia* [64].

**Figure 1:**
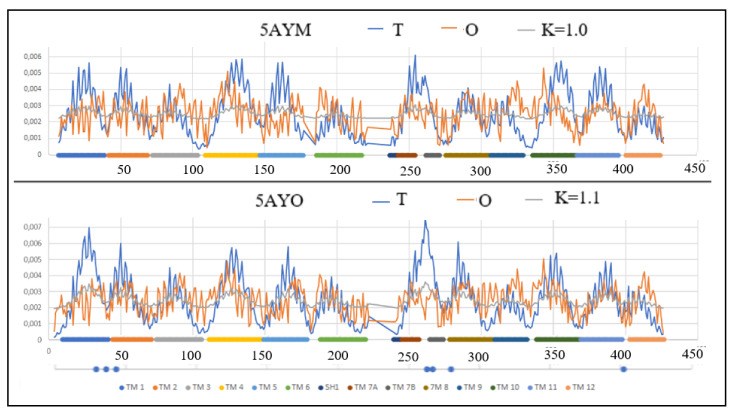
The profiles *T* (blue), *O* (red) and *M* (gray) for 5AYM and 5AYO. The value of *K* = 1 provides the appropriate degree of modification of the external field. Individual sections of TM were marked by different colors with identifiers assigned to particular local maxima. Profiles for 5AYO—bottom line—residues identified in [50] as engaged in vestibule interacting with substrate—discussed later in this paper.

The profiles *T*, *O* and *M* for the value of the parameter *K* = 1, which ensure an appropriate degree of modification of the external field, are shown in Figure 1.

The *T*-profiles of the proteins in question consist of 12 local maxima that correspond to the successive helices (denoted as TM) with sequential numbers. Almost all local maxima are not reproduced in the *O*-profiles, which means that the real (i.e., observed) distribution is different from the micelle-like pattern. The degrees of mismatch (described by the value of the parameter *RD* > 0.5) of individual sections (TM helices) present in the distribution *O* are given in Table 2.

The TM1 helix in In form compared to the Out one shows a much higher mismatch of the *O* distribution to the *T* (on the *RD* scale), which means its higher participation in the transporter structure. 

The status of TM2 helix (the second local maximum) turns out to represent a relatively high fit between *O* and *T* distributions in both discussed forms. This fact can be interpreted as the participation of this helix in the overall stabilization of the molecule and the lack of involvement in both the structure of the transporters and the contact with the membrane.

The status of TM3 helix in the compared structures, especially from the point of view of the adjacent loop, is different. In the case of the Out form, this loop shows a significant excess of hydrophobicity exposed on the surface of the molecule (the surface is identified as low value in the T distribution).

The TM4 helix reveals its contribution to the construction of the transporter in the form of Out (profile *O* is much lower than expected *T*). This helix, in the form of In, shows a significant agreement of the distributions of *T* and *O*. TM5 helix in the Out form shows a significant deficit of hydrophobicity, which means its participation in the structure of the transporter.

SH1 is the only helical segment oriented parallel to the membrane surface. It is located in the part exposed to the interior of the cell. The helix is present in the Out form. In the In form, as an independent helix with an orientation similar to SH1 (perpendicular to the system of TM helices), a helix 4 “also directed towards the interior of the cell is distinguished. The status of these helices is different. Fragment 4 is significantly more maladjusted to the expected distribution. It means a state of some collision with the aquatic environment inside the cell. In this respect, the form In is therefore in a disadvantageous state.

The TM6 helix is a section of the chain that is exposed on the surface of the molecule, and therefore is in contact with the membrane. The status of these segments in both forms turns out to be comparable, which proves a similar participation in the stabilization of TM6 in both forms of the discussed protein. However, the immediate vicinity of the polypeptide chain shows a significant mismatch in the Out form, exposing higher levels of hydrophobicity at the surface.

The TM7 helix in which two sections are distinguished shows a significant differentiation of each of its fragments, although the status of these sections in both forms is comparable. The 7A helix status contrasts with the 7B helix status showing a significant mismatch in the *T* and *O* distributions for the 7A helixes. However, by putting these two helices together (7A and 7B), they show a significant deficit of hydrophobicity, suggesting the presence of a transporter in their vicinity, while the 7B helix segment contributes to the overall stabilization of the structure.

The TM8 helix in the Out form shows a relatively high match of the *T* and *O* distributions in both forms. In the In form, the mismatch is significantly higher, indicating a significant differentiation of the *O* and *T* distributions. The N-terminal part in the In form shows a hydrophobicity deficit, so that in the C-terminal part of this helix it shows a significant excess suggesting participation in interaction with the membrane, thus stabilizing the location the whole molecule in this environment. As a result of this local variation, the TM8 status is described with higher values for both *RD* and *K*.

The TM9 helix in the Out form seems to be shifted with respect to the expectation. The local maximum in the *O* distribution is revealed in a different than expected location in the *T* distribution. The TM9 helix in the form of In shows a relatively higher adjustment of the *O* status to the expected *T*. The status of this TM10 helix in the Out form is significantly different than expected, which is expressed by a significant hydrophobicity deficit suggesting the participation of this helix in the transporter structure, which is not observed in the In form.

The status of TM11 helices turns out to be comparable for both forms of the discussed protein, showing relatively significant agreement of *O* and *T* distributions. The differential status of TM12 results from a certain shift of the *O* distribution compared to the *T* distribution in the In form. This can be interpreted as an alignment of the helical form elsewhere in the TM12 helix chain. The local maximum of hydrophobicity in this helix is expected to be in a different position than it actually is.

Comparing the In and Out profiles for 5AYO/5AYM reveals a change in the status of the first local high. The expected high maximum suggests the location of the amino acids in the central part of the molecule. A clear deficit reveals the presence of a transporter in the vicinity of the segment. In the case of the In form, the hydrophobicity deficit is clearly lower, indicating the presence of an open transporter in the vicinity of this helix. The relation of the *T* profile to *O* in the In version changes, revealing an approximation of the distribution of *O* to *T*. It does not mean restoring the centralization of the hydrophobic nucleus type, but reducing the divergence of the profiles *O* and *T*, which means reducing the free space in this region.

A similar interpretation applies to the heliacal segment 250–275, where also the divergence of the distributions is clear (smaller in the Out form). The opposite change can be observed for the helices 100–150 and 340–370, where the increased divergence of the distributions suggests the appearance of an open space in part of the transporter.

The status of surface sections is also changing. This is especially true of the section 300–350, where in the Out form the exposure of hydrophobicity is significant. This means a more favorable interaction with the surrounding cell membrane, which probably stabilizes the Out form to a greater extent.

**Figure 2:**
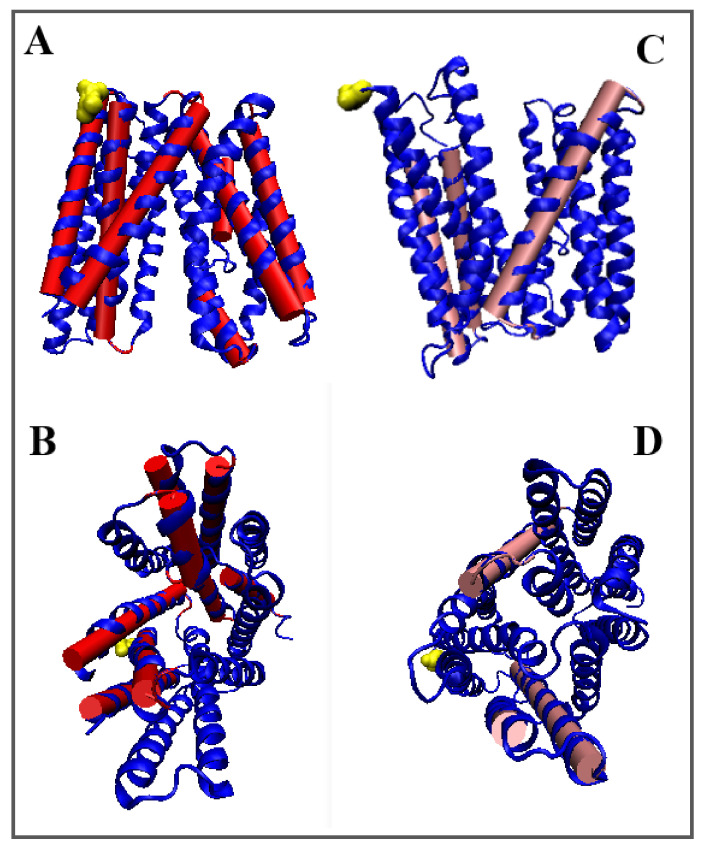
3D structures of In and Out forms with marked TM sections to show changed status changes, (**A**,**B**)—red 5AYM, (**C**,**D**)—pink 5AYO, Yellow—N-terminal residue to facilitate navigation. (**B**,**D**)—changed orientation.

In general, the comparison of the values of *RD* and *K* for all TM segments shows high convergence (expressed, for example, by the total value of parameter *RD*—for the helices in question *RD* = 3.13 for the Out form and *RD* = 3.42 for the In form). The total value of the *K* parameter is identical for both forms. This means that the system works in an alternating system, where overall Out state is equivalent to the In state. Only the participation of different fragments of the chain in the construction of the active form of this transmembrane transporter is changed. The 3D structure of TM helices with altered status in In and Out form (distinguished as bold in Table 3) are shown in Figure 2.

**Figure 3:**
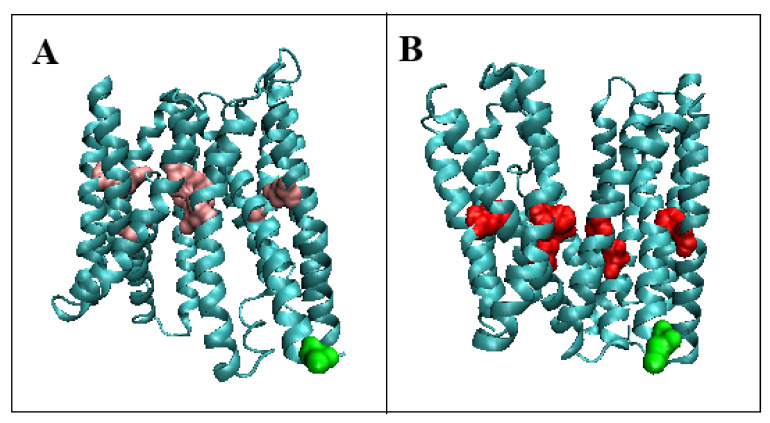
The change of the location of the expected center. Marked residues: (**A**)—Outward form (pink); (**B**)—Inward form (red). Green residues—N-terminal position for easy navigation.

Figure 3 visualizes the displacement of the location of the expected concentration in the form of a core.

The distribution of the maxima in the profiles indicates areas with high values of theoretical hydrophobicity *T* and relatively high values of the observed hydrophobicity *O*, which suggests the presence of a hydrophobic core. Figure 1 visualizes the change of its position depending on the form.

For the In and Out forms of the symport category, it is important to determine the status of the residues in contact with Fe^2+^ ions. The available structure of the Out form is a complex with Fe^2+^, while the Out form complexes K^+^ ions. The status of the residues involved in the interaction with Fe^2+^ ions in the Out form is *RD* = 0.773, while for the In form (the same residues are complexed by K^+^ ions), this status is expressed by *RD* = 0.751. If we take into account the immediate environment, i.e., the positions of the residues at a distance of ±5, this status is *RD* = 0.776 and 0.773, respectively. This means that the presence of ions does not have a significant influence on the status of the helices containing the residues interacting with the corresponding ion (helices 1 and 6).

Summarizing the conclusions resulting from the comparative analysis of *T* and *O* profiles of Out and In forms, the role of particular TMs can be characterized as follows. The structure of the transporter in the In form consists mainly of TM1, TM7A and TM7B helices, while in the Out form, these roles are played by the following helices: TM4, TM4′, TM5, TM7A and TM10.

A comparable and relatively consistent (*O* to *T*) distribution is represented by the TM2, TM8 and TM11 helices, being a component of structure stabilization. The change in the form of interaction with the membrane and the change in status to the aquatic environment affects the TM3, TM4, TM5 as well as TM8 and TM9 helices. Excessive exposure of hydrophobicity occurs mainly in the loops connecting the said helices, involving terminal fragments of the corresponding helices in this change.

The indicated residues F29, L36, L43 and V263, A267, I280 and F402 as hydrophobically interacting to form the extracellular gate, and as in many other transporters, allow for separation of the extracellular vestibule from the intracellular and in occluding the substrate at the binding site—shown in [50] in the present work—reveal the status defined by the FOD model as hydrophobically deficit: F29, V263 and A267, the status of local (but unitary) excess of hydrophobicity for L36, I280, with a consistent level of hydrophobicity determined for L43 and F402 and surface location (Figure 1). Therefore, the suggestion given in [50] seems to be confirmed in the present analysis.

#### 3.1.2. Representative of Solute Carrier 17 (SLC17) 

This group of proteins is represented by the structures PDB ID **6E9N** (inward-facing) and **6E9O** (outward-facing). By the symport mechanism, this protein uses H+ ions to transport the sialic acid anions out of the lysosomes. The TM helices listed in Table 4 are consistent with the system proposed in [51].

**Figure 4:**
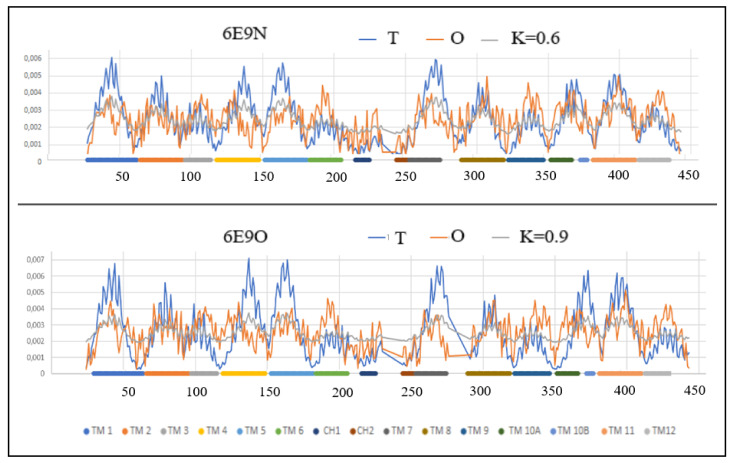
The profiles *T* (blue), *O* (red) and *M* (gray) for **6E9N** and **6E9O**. Individual sections of TM were marked with identifiers and differentiated by colors according to [51].

**Figure 5:**
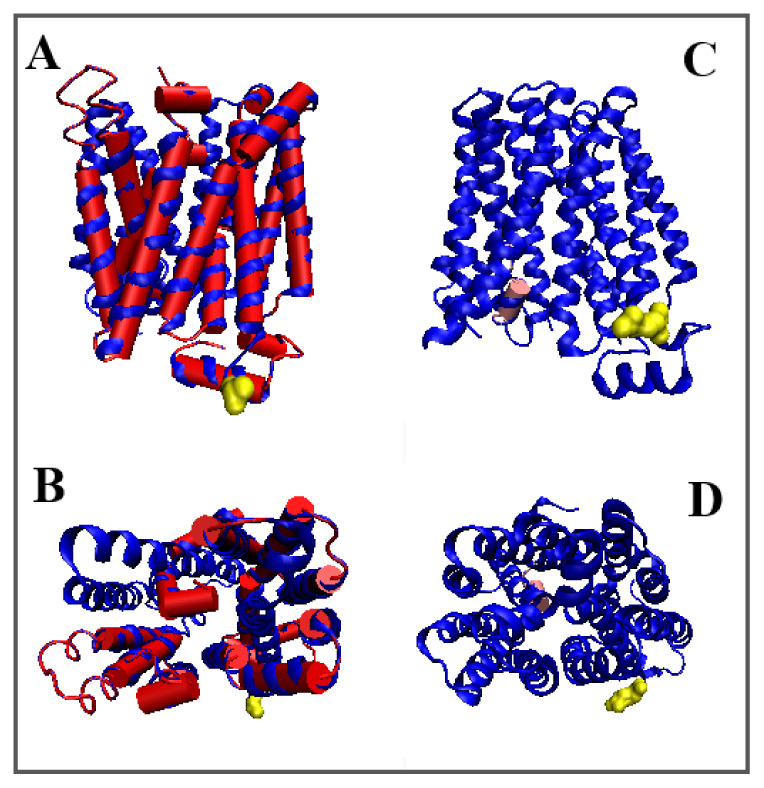
3D structures of In and Out forms with marked TM sections to show status changes, (**A**,**B**)—red 6E0O (**C**,**D**)—pink 6E9N, Yellow—N-terminal residue to facilitate navigation. (**B**,**D**)—different orientation od (**A**,**C**) presentation.

The overall assessment of TM helices shows a higher maladjustment in the Out form, where the total value of parameter *RD* = 9.57 for all helices (*RD* = 0.829 for that set in the In form) with a significant difference in the values of parameter *K* (*K* = 7.9/5.6 for the Out/In forms, respectively). This means that a higher deviation from the idealized hydrophobicity distribution is required for the Out form (Figure 4 and Figure 5).

The degree of packing of the form Out turns out to be higher than In—the local maxima reach values close to 0.007.

By carrying out an analogous analysis as before, the TM 3 and TM 4 sections can be identified as significantly more deformed in the Out form, which in the case of TM 4 means a higher participation of this helix in the transporter structure, while the changes in TM3 status result rather from the increased exposure of hydrophobicity on the surface in Out form. In the TM9 helix in the In form, the distribution of *O* is consistent with *T*—this is a factor in stabilizing the structure as a whole.

The TM 10A and 10B helices show a significant structural change resulting in an increased divergence of *O* and *T* distributions in the form of a significant hydrophobicity deficit in the Out form. This means the participation of this helix in the construction of the transporter in the Out form.

The summary assessment of the status of the In and Out forms indicates the presence of a significantly higher modification of the structure in the Out form, while the In form is closer to the structuring of the micelle-like order, based on the hydrophobicity distribution.

This match is higher but to the extent that is possible for a protein operating in the membrane environment, where the exposure of hydrophobic residues on the surface and a pronounced deficit in hydrophobicity in the central part of the protein caused by the presence of the transporter is evident for membrane proteins serving as a transporter. These standard deviations from the centric hydrophobic core determine the activity of membrane proteins.

Figure 5 presents the type and location of the helical sections identified with the FOD-M model changing their status during transport.

#### 3.1.3. Proton-Coupled Sugar Transporter Xy1E 

**Figure 6:**
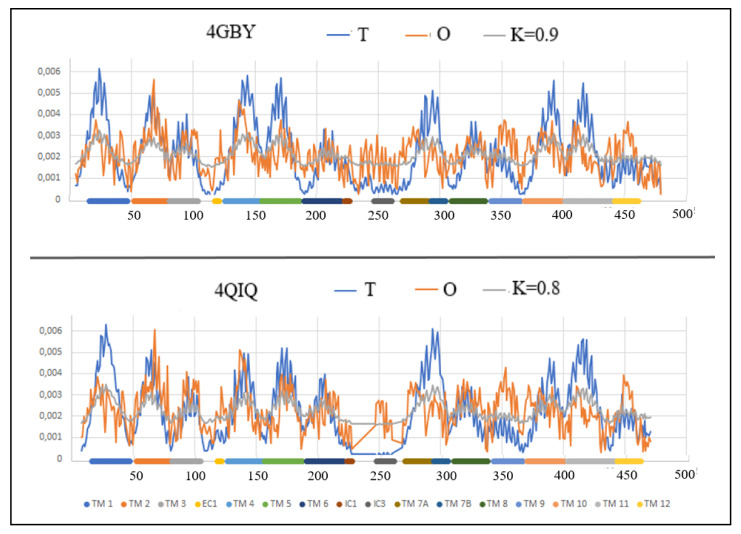
The profiles *T* (blue), *O* (red) and *M* (gray) for 4GBY and 4QIQ Individual sections of TM were marked with identifiers and color differentiation.

**Figure 7:**
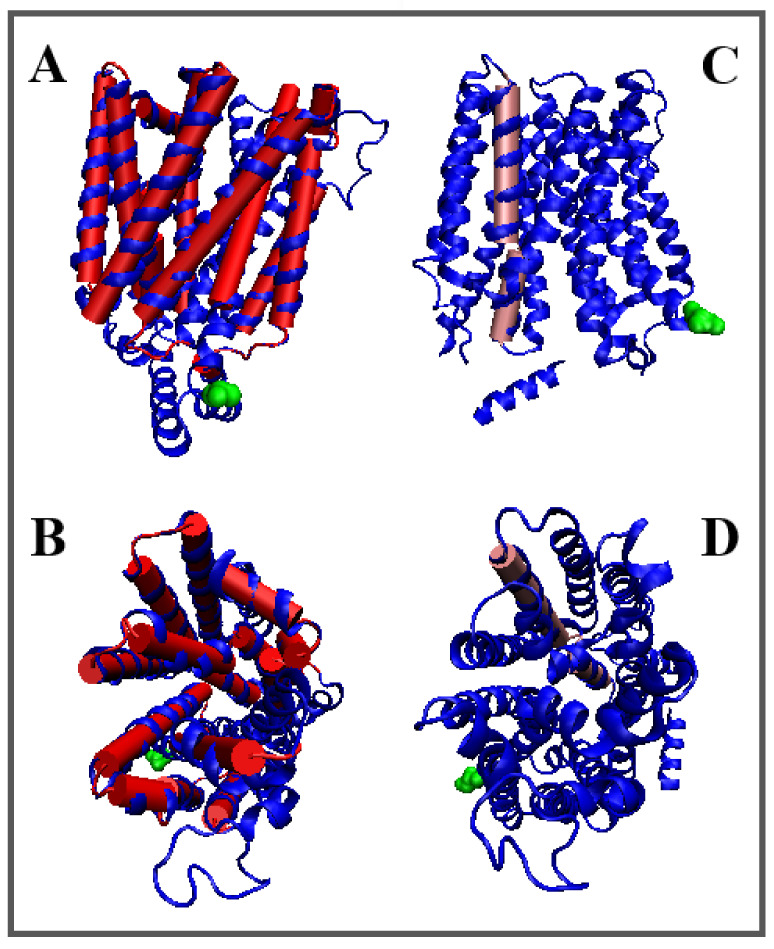
3D structures of In and Out forms with marked TM sections to show status changes, (**A**,**B**)—red 4GBY, (**C**,**D**)—pink 4QiQ, Green—N-terminal residue to facilitate navigation. (**B**,**D**)—different orientations shown in (**A**,**C**).

This protein is represented by the structures PDB ID 4QIQ (inward-facing) and 4GBY (outward-facing). The detailed analysis of the role of individual amino acids in the transport process, discussed in Refs. [52,53], is supplemented in the present work with the analysis of the status of individual TM sections of helices based on the hydrophobicity distribution (Table 5), which allows for an overview of the entire complex up-down bundle system (Figure 6 and Figure 7). 

#### 3.1.4. Multidrug Transporter MdfA

This protein, represented by the structures PDB ID 4ZP0 (inward-facing) and 6GV1 (outward-facing), belongs to the group Multidrug transporter (MdfA *Escherichia coli*) and performs in an antiport system. As can be seen from Table 6, presenting the status of individual helical sections, these statuses for both the Out and In forms show very similar values of the *RD* parameters, and the *K* value is even the same. The values of these parameters do not differentiate between these two forms.

If a protein transports two molecules at the same time in two opposite directions, it is difficult to define the form of Out and In unambiguously. It is necessary to determine for the transport of which molecules this status is defined. 

**Figure 8:**
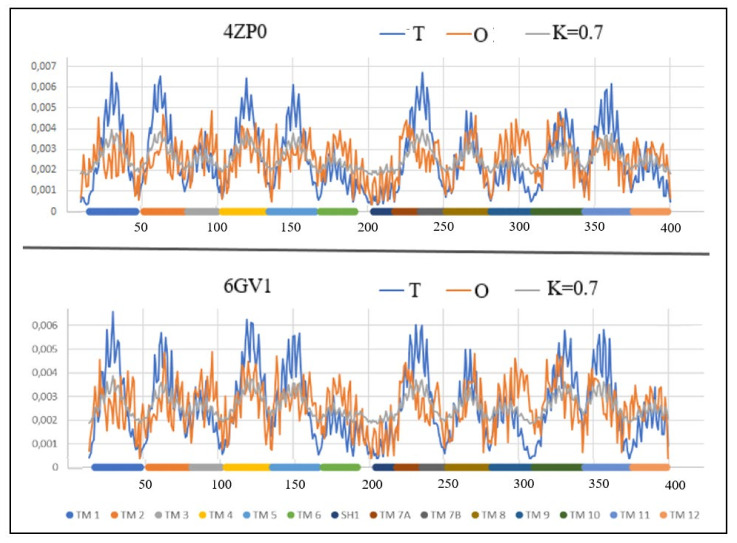
The profiles *T* (blue), *O* (red) and *M* (gray) for: A—2ZP0, B—6GV1. Individual sections of TM were marked with identifiers and color differentiation.

**Figure 9:**
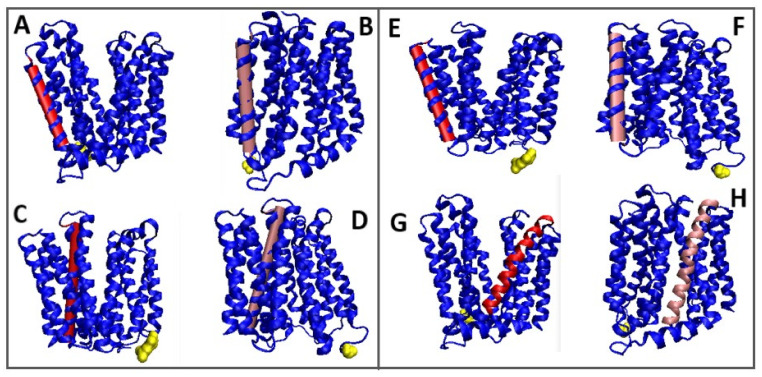
3D structures of In and Out forms with marked TM sections to show status changes, red 6GV1—pink 4ZP0, Yellow—N-terminal residue to facilitate navigation. (**A**,**B**): (sections 81–101); (**C**,**D**)—(section 218–251); (**E**,**F**)—(section 281–309); (**G**,**H**)—(section 343–375).

From the values of the parameters *RD* and *K* it can be concluded that the determination of Out and In is only contractual, since opening for both transport initiation and ending is comparable (Figure 8 and Figure 9). 

The items marked in Table 6 as bold identify those helical sections which, showing a status close to the system consistent with the micellar distribution, can be treated as sections stabilizing the whole structure. They are components of the part of the structure that meets the micelle-like ordering conditions, which expresses the magnitude of the influence of the aquatic environment on the final structure of the transmembrane protein.

Despite the very similar status of the Out and In structures, the total values of the parameters *RD* and *K* for Out/In forms is as follows: *RD*: 8.40/7.83 and *K*: 5.3/5.2, respectively. 

A 3D representation of the TM segments showing significant changes compared to In and Out forms is included in Figure 9. 

## 4. Discussion

The activity of proteins showing high specificity is determined by evolutionary changes in the amino acid sequence, which lead to the selection of a set of amino acids so that the structure “has tools” for carrying out appropriate biological processes. This applies to both local properties, such as the system of atoms/charges leading to the formation of a hydrogen bond or salt bridge, as well as global ones—for example, the creation of an appropriate external force field. For proteins operating in the aquatic environment, properly ordered polar water molecules provide a system of forces that guarantee the solubility of the molecule on the one hand, and on the other hand, create local, internal conditions enabling specific biological activity.

The cell membrane is the provider of the external field, completely different from the aquatic environment. In this field, the protein not only assumes the appropriate structure, but also undergoes strictly defined structural changes that are different from those that occur in proteins active in the aquatic environment [65].

The FOD-M model used in the present work, taking into account the presence and influence of an external force field, reveals the interdependence of structural changes related to function, and shows the participation in processes related to biological activity. This model can be an important tool for assessing the contribution of the external field to biological activity as well as to structuring. Protein folding in a non-aqueous environment has not been extensively analyzed, and even more so, dependence on the environment is very rarely present in the analyses. It often comes down to specifying the conditions of a given process, such as pH, ionic strength or the presence of other components. However, studying non-aqueous environments is important in the misfolding phenomenon [66]. The study of folding in a non-aqueous environment seems to be a great need to recognize the mechanism of protein folding [67].

The presented analysis provides information on transporter proteins, and in particular their form of Outward and Inward. Detailed analysis of the status of individual residues may provide material for researchers of the membrane transport mechanism. It is essential to include in the FOD-M model the presence of the aquatic environment. The description of the hydrophobicity distribution for membrane-anchored proteins was expected to be expressed as a function of 1-3DGauss. It turns out, however, that the proportion of water is present in the structuring of proteins completely embedded in the hydrophobic environment of the membrane. The presence of a hydrophobic membrane only modifies the influence of polar water on protein structuring. Quantitative determination of the proportion of water of the 1-st degree of its modification (parameter *K*) reveals the undeniable role of water as an environment for all life processes. The importance and role of the specificity of water as such is the subject of both experimental and theoretical analyses. Quantum chemistry techniques, in particular the DFT method, reveal a significant role of charge transfer in the structuring of water [68]. 

An overview of the current techniques for analyzing water as such and water as an environment for other processes is summarized in [69]. An important object of analysis is also the structure of water in the interphase: water-air, water-oil [70]. Some amino acids have been shown to influence the structuring of water [71].

To summarize the analysis based on the FOD-M model, a general principle should be given for the identification of residues remaining in contact with the membrane. These residuals are identified by the low values of the theoretical *T* distribution. A low value of *T* indicates locations on the surface of the protein. A high value of the actual (observed) *O* distribution for the same residue means the concentration of highly-hydrophobic residues, which should not be present on the surface in active proteins in the water environment. Examples include TM6, TM7B, and TM9 in 1GV1. This status can be read from the profiles (Figure 8).

## 5. Conclusions

The FOD-M model used for the analysis enables a comprehensive view of structural changes in transmembrane proteins (both symport and antiport) that perform transport functions through the cell membrane. The value added lies in the original assessment of the degree to which biological processes are influenced by the environment. This is accomplished through identifying the type of changes in the status of the helical sections that build the transmembrane protein. This type of analysis can complement the detailed analysis at the level of individual amino acids, or even individual atoms. The identified, differentiated participation of individual TM sections (in both types of transport) may reveal the specificity encoded in the discussed proteins and direct the research on the antibiotic resistance effect. The FOD-M model enables this type of analysis [60]. The applied model enables the identification and quantification of the structural deformation resulting from the change of the status and to relate them to the function.

The work reveals the role of hydrophobic interactions in the structural changes occurring due to the biological activity of proteins anchored in the environment of a hydrophobic membrane.

## Figures and Tables

**Table 1:** Characteristics of the discussed proteins. All structures were determined using the x-ray crystallography techniques.

Inward					Outward	
PDB ID [REF]	Chain Length	Transported Molecules	Transport	Source Organism	Chain Length	PDB ID [REF]
5AYO [50]	(4–426)	Proton/Fe^2+^	Symport	*Bdellovibrio bacterovorus*	(4–426)	5AYM [50]
6E9N [51]	(27–234) (243–443)	Proton/D-galactonate	Symport	*E. coli*	(24–230) (243–289) (295–442)	6E9O [51]
4QIQ [52]	(8–228) (247–471)	Proton/sugar	Symport	*E. coli*	(5–479)	4GBY [53]
4ZP0 [54]	(9–400)	Proton/antibiotic	Antiport	*E. coli*	(14–400)	6GVI [55]

**Table 2:** The values of the *RD* and *K* parameters calculated on the basis of the FOD-M model, describing the status of the discussed proteins (the values marked as bold indicate a higher degree of incompatibility between the *O* and *T* distributions.

INWARD			OUTWARD		
PDB ID	*RD*	*K*	*K*	*RD*	PDB
6E9N	0.596	0.6	**0.9**	**0.711**	6E9O
4QIQ	0.693	0.8	**0.9**	**0.697**	4GBY
5AYO	**0.725**	**1.1**	1.0	0.711	5AYM
4ZP0	0.647	0.7	0.7	0.642	6GV1

**Table 3:** The values of *RD* and *K* parameters for successive TM segments. TMs showing the status differences (increasing the value of *K*) in the compared forms In and Out are shown in bold.

HELIX		OUTWARD PDP ID—5AYM		INWARD PDB ID—5AYO	
TM	FRAGMENT	*RD*	*K*	*RD*	*K*
1	3–39	**0.772**	**1.5**	0.858	1.1
2	42–69	0.624	0.5	0.630	0.5
3	72–104	0.681	0.3	**0.610**	**0.5**
4	109–146	0.700	0.5	**0.835**	**0.7**
4”	138–146			0.844	0.4
5	147–177	**0.768**	**1.5**	0.462	0.8
6	186–218	**0.593**	**0.5**	0.580	0.4
SH1	237–242	0.557	0.1		
7A	243–255	**0.803**	**0.8**	0.805	0.6
7B	262–272	**0.414**	**0.2**	0.351	0.0
8	276–306	0.413	0.2	**0.647**	**0.6**
9	307–330	**0.759**	**0.8**	0.616	0.5
10	336–366	**0.758**	**0.9**	0.648	0.6
11	367–396	0.548	0.3	0.561	0.3
12	401–424	0.243	0.0	0.590	0.4

**Table 4:** The values of *RD* and *K* parameters for successive TM segments. TMs showing the status differences (increasing the value of *K*) in the compared forms In and Out are shown in bold.

HELIX		OUTWARD PDB ID—6E9O		INWARD PDB ID—6E9N	
TM	FRAGMENT	*RD*	*K*	*RD*	*K*
1	28–62	**0.683**	**0.5**	0.554	0.3
2	64–94	0.457	0.3	0.534	0.3
3	96–114	**0.800**	**0.8**	0.670	0.5
4	118–148	**0.891**	**1.9**	0.751	1.4
5	152–182	**0.738**	**0.5**	0.634	0.4
6	184–206	**0.578**	**0.4**	0.465	0.2
CH1	216–226	0.286	0.1	0.270	0.0
CH2	245–253	0.478	0.3	0.501	0.3
7	254–276	0.503	0.2	0.501	0.3
8	291–321	**0.537**	**0.4**	0.568	0.3
9	324–349	**0.714**	**0.7**	0.445	0.2
10A	354–369	**0.932**	**1.0**	0.661	0.4
10B	375–380	0.822	0.3	**0.681**	**0.6**
11	384–414	0.600	0.3	0.502	0.2
12	416–438	0.556	0.2	0.557	0.2

**Table 5:** The values of *RD* and *K* parameters for successive TM segments. TMs showing the status differences (increasing the value of *K*) in the compared forms In and Out are shown in bold.

HELIX		OUTWARD PDB ID—4GBY		INWARD PDB ID—4QIQ	
TM	FRAGMENT	*RD*	*K*	*RD*	*K*
1	16–47	**0.684**	**0.6**	0.672	0.5
2	52–80	0.356	0.2	0.505	0.3
3	81–104	**0.610**	**0.5**	0.308	0.0
EC1	118–121	0.385	0.1	0.473	0.1
4	126–155	0.419	0.1	0.457	0.2
5	156–187	**0.776**	**0.6**	0.475	0.2
6	190–220	**0.783**	**0.6**	0.642	0.5
IC1	223–228	**0.621**	**0.4**	0.382	0.1
IC3	247–261	0.349	0.1	**0.716**	**1.1**
7A	270–291	**0.899**	**1.6**	0.734	0.9
7B	293–305	**0.664**	**0.4**	0.535	0.2
8	310–337	**0.648**	**0.4**	0.560	0.3
9	342–366	**0.657**	**0.6**	0.523	0.3
10	369–399	0.885	0.9	**0.880**	**1.2**
11	402–440	**0.602**	**0.5**	0.625	0.4
12	442–461	0.425	0.2	0.455	0.2

**Table 6:** The values of *RD* and *K* parameters for successive TM segments. TMs showing the status differences (increasing the value of *K*) in the compared forms In and Out are shown in bold.

HELIX		OUTWARD PDB ID—6GV1		INWARD PDB ID—4ZP0	
TM	FRAGMENT	*RD*	*K*	*RD*	*K*
1	16–47	0.769	1.2	0.763	1.2
2	52–80	0.676	0.5	0.693	0.5
3	81–101	**0.506**	**0.2**	0.362	0.1
4	104–134	0.514	0.3	0.577	0.3
5	135–166	0.723	0.6	0.685	0.6
6	169–192	0.308	0.1	0.331	0.2
SH1	204–217	0.415	0.2	0.283	0.1
7A	218–234	0.597	0.3	**0.660**	**0.5**
7B	234–251	**0.781**	**0.8**	0.878	0.6
8	251–281	0.427	0.2	0.276	0.1
9	281–309	**0.760**	**0.7**	0.628	0.4
10	309–343	**0.641**	**0.4**	0.509	0.2
11	343–375	0.629	0.4	**0.557**	**0.8**
12	375–399	0.660	0.6	**0.630**	**0.8**

## Data Availability

All data can be available on request when addressed to the corresponding author. The program allowing calculation of *RD* is accessible on the GitHub platform: https://github.com/KatarzynaStapor/FODmodel (accessed on 15 September 2022) and on https://hphob.sano.science (accessed on 15 September 2022).
